# Knowledge brokers, companions, and navigators: a qualitative examination of informal caregivers’ roles in medical tourism

**DOI:** 10.1186/1475-9276-12-94

**Published:** 2013-12-01

**Authors:** Victoria Casey, Valorie A Crooks, Jeremy Snyder, Leigh Turner

**Affiliations:** 1 Department of Geography, Simon Fraser University, Burnaby, British Columbia, Canada; 2 Faculty of Health Sciences, Simon Fraser University, Burnaby, British Columbia, Canada; 3 Center for Bioethics, School of Public Health, and College of Pharmacy, University of Minnesota, Minneapolis, Minnesota, USA

**Keywords:** Informal caregiving, Medical tourism, Global health services, Healthcare providers, Qualitative analysis, Family caregivers

## Abstract

**Introduction:**

Many studies examining the phenomena of medical tourism have identified health equity issues associated with this global health services practice. However, there is a notable lack of attention in this existing research to the informal care provided by the friends and family members who typically accompany medical tourists abroad. To date, researchers have not examined the care roles filled by informal caregivers travelling with medical tourists. In this article, we fill this gap by examining these informal caregivers and the roles they take on towards supporting medical tourists’ health and wellbeing.

**Methods:**

We conducted 21 interviews with International Patient Coordinators (IPCs) working at medical tourism hospitals across ten countries. IPCs work closely with informal caregivers as providers of non-medical personal assistance, and can therefore offer broad insight on caregiver roles. The interviews were coded and analyzed thematically.

**Results:**

Three roles emerged: knowledge broker, companion, and navigator. As knowledge brokers, caregivers facilitate the transfer of information between the medical tourist and formal health care providers as well as other staff members at medical tourism facilities. The companion role involves providing medical tourists with physical and emotional care. Meanwhile, responsibilities associated with handling documents and coordinating often complex journeys are part of the navigation role.

**Conclusions:**

This is the first study to examine informal caregiving roles in medical tourism. Many of the roles identified are similar to those of conventional informal caregivers while others are specific to the transnational context. We conclude that these roles make informal caregivers an integral part of the larger phenomenon of medical tourism. We further contend that examining the roles taken on by a heretofore-unconsidered medical tourism stakeholder group sheds valuable insight into how this industry operates and that such knowledge is necessary in order to respond to the health equity debates that surround this particular global health services practice.

## Introduction

Health care delivery involves many provider groups, both formal and informal, that address different facets of patient care. Doctors and nurses are examples of formal provider groups in that they receive specialized training specific to the care they deliver, are paid to deliver this care, and are commonly licensed professionals [1,2]. Much social health research has documented the important and complex responsibilities that formal providers assume in ensuring patients’ health and general wellbeing e.g. [3-5]. In contrast, informal health care providers include friends, family, and some volunteers. Members of these groups provide essential care despite the fact that they are not formally trained and paid health professionals [6,7]. These informal care providers, or caregivers, often provide ongoing essential care in the home, such as administering medications, managing wounds, and assisting with rehabilitation [8]. Meanwhile in hospitals and other types of residential care settings, they perform tasks that augment services provided by on-site formal providers. Examples of such tasks include monitoring symptoms and articulating patients’ preferences to health care professionals [9-11]. The roles that formal and informal providers fill and the responsibilities assigned to each collectively work together to enable health care delivery. In this article, we examine the caregiving roles assumed by a specific group of informal health care providers: the friends and family members that accompany medical tourists abroad for private medical care.

Hospitals, clinics, and individual health care practitioners who provide medical treatment to privately-paying traveling international patients who do not have a formal cross-border care referral are part of what is often described as the ‘medical tourism industry’ [12-14]. This industry is reported to be on a multi-billion dollar scale and involves the transnational movement of patients to a growing list of destination countries located on nearly every continent [13,15-18]. There are a great variety of procedures that can be obtained in medical tourism facilities, including, though not limited to, cosmetic surgeries, cardiac surgeries, and orthopedic surgeries [15,19,20]. Significant concern has been raised that the medical tourism industry is exacerbating health inequities in destination countries through such measures as recruiting formal health care providers out of public systems and into private ones, which prices locals out of needed health care [21]. Supporters of the industry meanwhile suggest that the practice of treating privately-paying international patients can assist with addressing health inequities through measures such as retaining formal providers who may otherwise have left the country in search of higher pay or more technologically sophisticated practice and bringing in capital [21].

Research examining medical tourism has paid some consideration to numerous health care provider groups central to this global health service practice. Perhaps not surprisingly, much of this research examines formal health care providers rather than informal ones, though sometimes members of the latter category are mentioned in passing. Although academic studies and industry reports note that it is common for medical tourists to be accompanied by friends and family members (e.g., [6,16,22-24]), they are rarely framed as informal health care providers or as a stakeholder group meriting additional exploration. Meanwhile, it is widely acknowledged that informal caregivers provide the bulk of patient care throughout the life course and across the care continuum and play an important role in establishing care recipients’ health outcomes [7,24-26]. Given the importance of informal caregivers in health care provision, it is surprising that their contributions to the care of medical tourists have not attracted greater scrutiny. Only non-academic work has addressed the practice of informal caregiving in medical tourism. Two published narratives of medical tourists’ journeys written by their informal caregivers reveal that significant caregiving responsibilities were assumed by these individuals [27,28]. Their detailed narratives document numerous instances of making decisions on behalf of the patient, liaising with formal providers, coordinating appointment scheduling, offering hands-on care, providing emotional and spiritual support, and taking responsibility for managing care-related finances. These narratives, although not scholarly in nature, are nonetheless illuminating because they suggest that informal providers can play a significant role in the practice of medical tourism. In this article, we aim to identify and explicate the informal caregiving roles that friends and family members assume in the course of a medical tourist’s journey. By ‘roles’ we refer to their key social functions in the maintenance of medical tourists’ health and wellbeing and the larger transnational care practice of medical tourism.

Our exploration of informal caregivers’ roles herein is informed by insights gleaned from interviews with International Patient Coordinators (IPCs) working at medical tourism facilities. IPCs work at destination facilities; their task is to coordinate medical tourists’ care. Their responsibilities include arranging ground transportation and local travel, communicating with doctors, scheduling medical appointments, and providing support and guidance for patients and their caregivers. Because of the nature of their jobs, every year they interact with anywhere from tens to hundreds of medical tourists and their informal caregivers. Given their function, we believe that by sharing their observations and experiences they are well positioned to identify the informal care roles filled by this caregiver group. In the section that follows we provide an overview of the study design and a description of the 21 IPCs with whom we spoke. We then present the findings of a thematic analysis that identified three roles commonly filled by medical tourists’ informal caregivers: knowledge broker, companion, and navigator. We subsequently discuss the findings in light of the existing medical tourism and informal caregiving literatures and offer directions for future research. We conclude by reflecting on the relevance of this analysis for providing new insights that have relevance for the health equity debates that surround the global medical tourism industry.

## Methods

This analysis emerges from a large, multi-method study that explores first-hand accounts of medical tourists’ informal caregivers and those who have worked closely with them in a professional capacity. Here, we report on the findings of interviews conducted with IPCs about their interactions with and observations of these caregivers. The findings speak to the roles that caregivers from a range of home countries fill as they accompany medical tourists seeking a variety of medical procedures at international health care facilities.

IPC recruitment commenced upon receiving approval from the Research Ethics Board at Simon Fraser University. We sought participants from a diverse range of countries and facilities using several concurrent methods: (1) emailing letters of invitation to hospitals and clinics whose websites mentioned IPCs, IPCs identified in online medical tourism directories, and IPCs who had posted on online forums; (2) snowballing out from initial participants; and (3) disseminating calls for participants through our team’s networks and online medical tourism industry forums and magazines. Recruitment materials indicated that interviews could be conducted in English or French. A later request for a Spanish-language interview was also accommodated.

Interested potential participants who contacted us by e-mail were sent an information sheet that provided additional information concerning the study and described their rights as participants including confidentiality. Before this sheet was sent, participant eligibility was confirmed. Because many potential participants did not use ‘IPC’ as their official job title, they were required to indicate that: they worked with international patients who obtained procedures at medical tourism hospitals or clinics that offered surgical procedures without third party involvement such as organ transplantation; they were physically present in the facility with the medical tourist; they made care and other arrangements; and they assisted clients in a non-clinical capacity. To capture diversity among the sample, no more than three individuals from a single facility were interviewed. We stopped active recruitment and interviewing when we reached our target sample size of 20 interviews, a point that coincided with when new potential participants were no longer being identified.

Interviews were conducted over telephone or Skype according to the participant’s preference. They typically lasted for 45-75 minutes. A semi-structured interview guide was used, enabling the capture of issues central to the study’s objectives and topics that were important to the participants. The first author conducted 19 English-language interviews while a knowledgeable collaborator conducted one in Spanish. No French-language interviews were requested.

Verbal consent was obtained before each interview, and the interviews lasted for approximately 45-60 minutes. As shown in Table 1, the interviews covered topics such as: (1) informal caregiver characteristics, (2) interactions between caregivers and medical tourism facility staff, (3) caregivers’ roles and responsibilities, and (4) the risks to which caregivers can be exposed while travelling with medical tourists and providing care to them.

**Table 1:** Selected interview questions

**Question**	**Sub-probes**
In your experience, what is the typical relationship between patients and their travel companions?	What are some of the common characteristics of travel companions?
What are some of the reasons that you interact with travel companions?	Before arrival? While abroad? Upon returning home?
What kinds of responsibilities do you commonly see travel companions taking on?	In relation to: communication; symptom monitoring; hands-on care; corresponding with friends and family at home; providing emotional support to the patient; providing spiritual support to the patient; making arrangements; travel and tourism activities; other relevant activities?
Have you ever experienced a situation where a travel companion’s health worsened or improved while they were abroad (both in hospital or after discharge)?	How common is this? What could be the cause of their worsened or improved health? Can you think of any problems, stresses, difficulties that travel companions face while the patient is in hospital and after discharge?

Twenty interviews were conducted with 21 IPCs (one interview had two participants) and drew from their experiences working at 16 different medical tourism hospitals or clinics in Bolivia, Costa Rica, Barbados, Mexico, the United States, Croatia, India, Israel, Thailand, and Turkey. Twelve IPCs mainly dealt with North American medical tourists, six mostly serviced Europeans, one primarily saw Australians and another Africans, and the remaining two did not report a particular regional orientation. The procedures provided at the facilities where the participants worked included cosmetic surgery, bariatric surgery, orthopedic surgery, oncology procedures, spinal surgeries, veinoplasty, and cardiac surgery.

All interviews except one were recorded digitally and transcribed verbatim. Technical difficulties prevented the exception from being recorded, and detailed interviewer notes were instead used to document the interview. All transcripts and notes were loaded into NVivo, a qualitative data management program, after which thematic analysis was conducted.

The thematic analysis involved six steps. First, all investigators reviewed the transcripts and notes. Second, emerging themes and outliers were identified during a face-to-face meeting with all investigators. Third, the first and second authors created a preliminary coding scheme that identified overall thematic concepts and their components. This involved collaboratively creating tiers of category headings that were manipulated until the authors were confident that they would entirely capture the study objectives as well as the themes and outliers identified in the meeting. Fourth, the first author coded the data in NVivo, with input on code refinement and interpretation from the second author. Fifth, the first and second authors identified emerging trends and patterns relevant to the themes pursued in the current analysis, namely those pertaining to caregivers’ roles. Sixth, a refined interpretation of meaning in the coded data was revealed through a comparison of the trends and patterns with existing knowledge and the study objectives [29]. This comparison was initially done by the first and second authors and then confirmed by the full team. Characteristic of thematic analysis, this analytical process enabled common themes to emerge despite the differences in IPC participants’ work environments and work histories [30].

## Results

**Figure 1:**
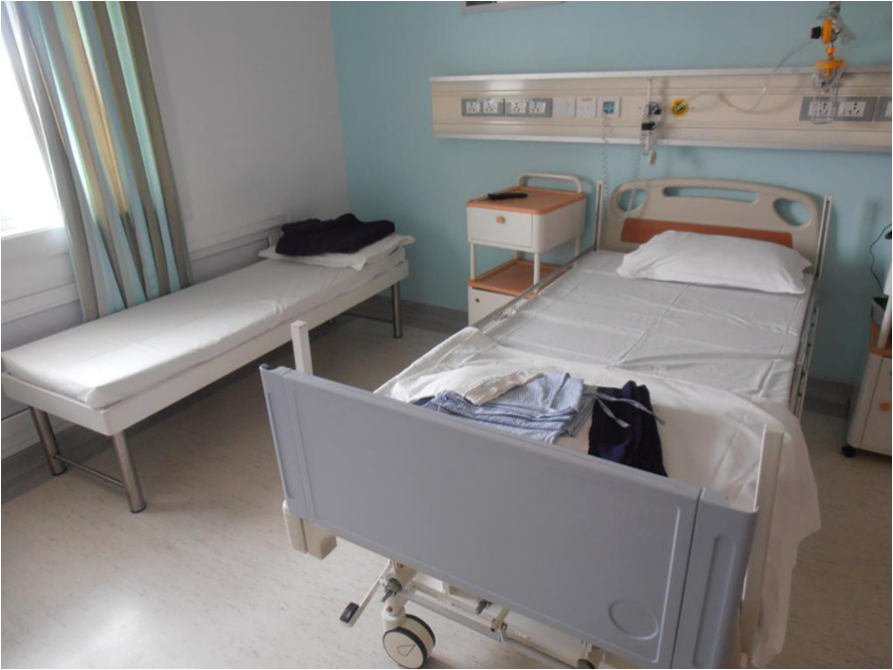
**Typical medical tourist patient room.** Taken at hospital in India that treats medical tourists, this photo conveys the close physical proximity that is experienced between some medical tourists and their informal caregivers while abroad. The cot on the left is for the friend or family member providing care while the bed on the right is for the patient. (Photo credit: authors).

All IPCs reported that it was common for medical tourists to bring at least one friend or family member abroad with them unless they were specifically discouraged from traveling with a companion^a^. Family members, especially spouses, were the most common type of informal caregivers present at the facilities where participants worked. Facilities that offered in-patient procedures often had cots or beds available in patients’ rooms for these informal caregivers. This arrangement demonstrates the intense physical proximity of this transnational caregiving practice. Figure 1 provides an example of this kind of patient room. In the case of out-patient clinics or where co-habitation in the patient’s hospital room was uncomfortable or impossible, friends and family typically stayed at nearby hotels or rented apartments.

Thematic analysis of interviews conducted with 21 IPCs about their interactions with and observations of medical tourists’ informal caregivers revealed three roles commonly adopted by this group: (1) knowledge broker, (2) companion, and (3) navigator. The knowledge broker role refers to key functions undertaken by caregivers around the transfer of information between the medical tourist and facility staff or others. The companion role refers to functions around the provision of emotional and physical comfort or support as well as hands-on care. Finally, the navigator role encompasses functions dealing with information gathering and care coordination responsibilities assumed by medical tourists’ informal caregivers. While IPCs observed that many caregivers adopt all three roles, often simultaneously, we consider them separately in this section in order to provide detailed accounts of the distinct features of each role. We include verbatim quotations throughout in order to ‘give voice’ to the participants. Each quotation is followed by a country name and a number. The name refers to the country in which the participant worked at the time of the interview while the number indicates the number of years they had worked as an IPC at that particular facility.

### Knowledge broker

Participants emphasized that a key role caregivers play is that of knowledge broker. The facilitation of knowledge transfer from medical tourism facility staff to the patient by caregivers is typically done in four ways: inquiry, clarification, translation, and retention. First, caregivers commonly make inquiries on behalf of patients. Questions directed toward IPCs tend to be about logistics or planning, while those directed at formal providers cover clinical concerns such as prescriptions, care options, and advice for after discharge. Second, by seeking clarification caregivers help to ensure that patients clearly understand medical information. This activity can be especially helpful when patient rights are explained, when complications occur, or when patients are confused about what they have been told. Third, caregivers help with translation or basic communication when they are more proficient than the patient in the language or regional vernacular used by facility staff. “*When the patient comes over here, the language may differ or the accent may differ, [the understanding of] English between the patient and the [facility] staff may differ slightly, so there needs to be someone who can patiently communicate with the [facility] staff [if the patient cannot]*” (India, 2.25). Finally, caregivers retain information that has been conveyed by facility staff to the patients. “*Sometimes when you’re in that position [as a patient], you’re ill, you’re in a bed, the doctor comes in and says x, y or z, you don’t remember it… So it’s nice to have someone there with you who will be able to retain that information*” (Barbados, 2). Knowledge brokering by facilitating the transfer of information from facility staff to patients can be demonstrative of collaboration between caregivers and formal providers and other facility staff.

Caregivers often engage in knowledge brokering by offering unprompted but useful information about medical tourists to facility staff. IPCs noted that caregivers frequently offer this kind of information while patients are at the facility and also following discharge. For example, caregivers may voice patients’ concerns or articulate complaints when patients are reluctant to make such remarks. They may also help formal providers obtain correct information when patients are untruthful (e.g., purposely not reporting their correct weight), do not accurately recall their health history, or are unwilling to communicate. For example, one participant reported that she once worked with a patient who was uncommunicative and therefore the accompanying caregivers answered her questions instead: “*…sometimes the patient didn’t feel like talking and then his mother or his wife would give me the information*” (Mexico, 3). These types of information exchanges between facility staff and caregivers, ones that are not prompted by the medical tourist and may not occur in the patient’s presence, reveal *“…the good, the bad, the ugly, everything [about the patient that may be helpful to facility staff]*” (Costa Rica, 2.5). In other words, caregivers may broker information or share details with facility staff to ensure that they have accurate information with which to make decisions that affect patients’ health independent of requests to do so from medical tourists.

Participants observed that caregivers often act as information liaisons between patients and friends and family members back home. They provide friends and family members with updates by phone, email, Skype, and even letters. One participant recounted that caregivers commonly “*bring their computer [or] they can borrow our telephones and right after the surgery they…communicate with people from home just to let them know that everything went okay*” (Mexico, 3). Acknowledging the importance of this aspect of caregivers’ roles, some of facilities even offer them free international calling. This dimension of knowledge brokering serves to underscore the truly transnational nature of the roles that caregivers take towards medical tourists as well as within the larger practice of medical tourism.

### Companion

One way that informal caregivers engage in the companion role is by creating a feeling of emotional safety and security for the medical tourist. A participant reported that caregivers provide “*… emotional support, which was probably the most crucial [thing that they can do]. It’s something that a medical tourism company cannot offer*” (Turkey, 1.5). Caregivers are familiar to patients, they have established relationships with one another, and bonds of trust already exist before their journeys begin. These traits put them in a unique position to offer better quality support, including moral and spiritual, relative to facility staff. Caregivers’ trusting relationships with medical tourists often makes them aware of patients’ preferences and needs. All the IPCs with whom we spoke indicated that trusting relationships between patient and caregiver can have emotional benefits for the medical tourist and that this social bond is a significant reason why they encourage medical tourists to avoid travelling alone. To a medical tourist, a caregiver is “*someone there who would be able to share your…experience…you wouldn’t have to…be looking for words, you would just be able…to flow with this person because this is someone that you know, it’s someone you have a history with, so you’d be more comfortable*” (Barbados, 2). According to the participants, the presence of the caregiver can be beneficial to creating a feeling of safety and security for the patient.

Many participants noted that caregivers typically try to address medical tourists’ comfort in their role as companions. This assistance comes in the form of: providing the patient with wanted or needed items, monitoring symptoms, and helping the patient deal with the ‘foreignness’ of the destination country. IPCs commonly observed caregivers obtaining items for medical tourists inside and outside the facility because, participants postulated, patients were more comfortable asking their companions than their IPC. For example, patients who dislike the food served at the facility can send caregivers into the surrounding area on regular food purchasing trips. To ensure physical comfort, nearly all participants noted that caregivers monitor patients’ symptoms and alert formal providers of noteworthy changes. A participant explained: “*…we provide them with our extension numbers and they might call us just to say ‘…my wife is having nausea after the operation, do you think somebody can give her some medicine’. So we do quite often see that situation*” (Thailand, 6). Caregivers may further ensure patients’ comfort by being a familiar, reliable figure in an otherwise unfamiliar environment. IPCs explained that being outside one’s own country can be inherently stressful. Language differences in particular can be especially difficult for the patient. Patients and caregivers “*really just have each other… I mean the outside world speaks a different language, different culture. They rely a lot on each other*” (Turkey, 1.5).

Many participants reported that caregivers commonly provide hands-on care, especially after the patient is discharged from the facility. This care may take the form of assistance with mobility and everyday tasks and help with following clinical advice. Patients’ mobility and their capacity to perform everyday tasks after surgery are typically minimal, and therefore some facilities require that medical tourists bring a caregiver to assist them with daily activities following the operation. Caregivers often help patients with dressing, showering, toileting, and mobility at the facility and after discharge. After discharge “*… patients…might need the help of a companion with luggage, with getting in and out of a vehicle, perhaps getting up to their hotel room, go out to dinner, things like that*” (USA, 8). Caregivers also help patients follow clinical advice such as taking medications, sometimes offering hands-on care to ensure this advice is followed. One participant explained that caregivers need to know “*what not to do [when caring for the patient]: do not…bend the knee over this position if he has a knee replacement, or do not give him anything to eat besides what the doctor says if he has a gastric sleeve…*” (Costa Rica, 0.6). In some cases, caregivers continue this aspect of their companionship role after returning to their home country, ensuring appropriate post-operative and follow-up care regimens are followed.

### Navigator

Caregivers fill a navigator role when they guide the patient through various aspects of the medical tourism experience. One such aspect is geographical and cultural navigation. For example, caregivers typically gather tourist information. As mentioned above, they may do this by asking facility staff, especially IPCs, questions about particular destinations in the local area. Some of these questions may pertain to tourist activities that are suitable for the patient after discharge. Caregivers may also seek location-specific information from IPCs, such as “*how to take a taxi and…what areas are safe to go to and what aren’t…*” (Mexico, 12). In addition to gaining familiarity with navigating the destination country or city, caregivers typically learn how to navigate the destination facility and transmit this information to the medical tourist. According to one participant, caregivers are “*the ones who read through the instructions [about the facility] and enforce them [with the patient]…*” (Mexico, 1.5, 0.6). Participants also noted that, in their capacities as navigators, all caregivers familiarize themselves with useful locations in the facility such as bathrooms and magazine vendors.

Most participants observed that a large part of a caregiver’s navigator role involves coordinating paperwork and gathering required documentation. “*Usually the companion, to relieve the patient that’s having the surgery, does all the running around to make it happen*” (Costa Rica, 5). Many IPCs reported that completing paperwork is one of the first tasks that caregivers must undertake after arriving at the facility. This task requires completing forms for the patient and verifying that patient information is accurate. They also “*…tend to want to deal with the finances…they’re always very worried about [the patient] being worried about…the balances or the costs that their stay incur. They tend to want to shield [the patient] from that*” (Barbados, 2). They typically monitor and complete financial paperwork and may access a bank in person or electronically to exchange currency, to ensure funds are available, and to get money for airfare, treatment, and other expenses. Caregivers also commonly transfer documentation from the facility abroad to patients’ regular physicians and vice versa before departure and upon return home. These documents include medical records, letters, prescriptions, and/or test results. IPCs remarked that navigating the coordination of paperwork and documentation is generally done by caregivers to minimize the number of concerns or stressors that medical tourists encounter.

Creating plans and ensuring that travel itineraries are followed are both common responsibilities for caregivers in their roles as navigators. Caregivers sometimes play a large role in preparing for the trip abroad. They communicate with IPCs to organize the trip, sometimes as early as sending the initial inquiry. They continue to fill this role throughout the stay by arranging lodging, tours, and food options. They may further help patients by arranging ground transportation and international flight arrangements. “…*They get the wheelchair for them and, you know rent a chair car to transport the patient to and from the hotel…*” (USA, 8). Caregivers also sometimes create itineraries with the help of IPCs, and typically ensure that these plans are followed by the medical tourists. A key reason for this is because medical tourists typically look to caregivers “*for [information about] the whole journey, you know: ‘What time are we going to the doctor? What time is our appointment? What time is the check-up? When do we go next? Tomorrow’s a day off, are we going touring? Are we going to the beach? Are we going shopping?’*” (Israel, 3). Caregivers know the estimated length of the patient’s stay in the facility and note changes in the recovery schedule after surgery so that they can alter or cancel activities as needed. Even after they are back in their home country, caregivers often help patients create and follow an itinerary of follow-up appointments. The majority of participants noticed that caregivers generally help patients navigate their experience abroad by making and altering plans as needed, and that nearly all of them play an important role keeping patients on schedule.

## Discussion

The analysis has found that medical tourists’ informal caregivers adopt three major roles that complement those of formal health care providers: knowledge broker, companion, and navigator. As knowledge brokers, they facilitate the transfer of information between the medical tourist and the formal health care providers, the IPC, friends and family members back home, and others. As companions they support the medical tourist emotionally by ensuring their comfort and providing hands-on care. Finally, in their role as navigators they gain familiarity with the destination country, coordinate the trip, and handle facility documents. In this section, we examine interconnections between these roles and consider all three in light of the existing medical tourism and informal caregiving literatures. We also identify future directions for research emerging from the findings.

Each of the three caregiver roles identified by IPCs encompass several responsibilities. Many responsibilities are unique to a single role. Handling finances, for example, which is part of the navigation role, has no overlap with other role components, though the outcomes of this responsibility might have implications for caregivers’ other roles. Other responsibilities might seem less clearly delineated with regard to the roles to which they contribute because of the similarities of the activities they involve. For example, there are information exchange aspects to all the roles. The difference is the intermediary or primary position of the caregiver in the exchange and the nature of the information. Offering or retaining information on behalf of the patient is characteristic of the knowledge broker role. Meanwhile, flagging the patient’s changing health status to staff relates most closely to the companion role, whereas discussion about travel logistics relates to the navigator role. By both focusing on the intent of an action being undertaken and having identified the scope of each role it was clear in the analytic process as to which responsibilities were attributed to what roles.

A strong relationship exists between the caregiver roles because the actions, activities, and overall responsibilities undertaken in one role can have implications for the other two roles. In this way, there is overlap between all the roles identified in the findings. By helping patients follow clinical advice, which pertains to the companion role, caregivers act on advice they might have retained in their capacity as a knowledge broker. When the clinical advice requires arranging medical appointments and ensuring that patients arrive at appointments in a punctual manner, then there is also overlap with the navigator role. Knowledge brokering and the companion role further overlap when caregivers monitor medical tourists’ symptoms and communicate those observations to a health care provider; symptom monitoring is identified as part of the companion role, whereas voicing comments or concerns about symptoms to formal providers is part of the knowledge broker role. These are but a few of the many examples of the ways that distinctive roles become interconnected through the practice of informal caregiving in medical tourism. Although these interconnections can create some overlaps or redundancies between roles, we believe that the distinctions between the knowledge broker, companion, and navigator roles remains useful for clearly positioning the friends and family who accompany medical tourists abroad as informal caregivers, and ultimately unpaid health care providers, within the industry.

Whereas the existing medical tourism literature does not discuss informal caregivers’ roles in detail, there are some mentions of the same activities or responsibilities discussed by the IPCs with whom we spoke. Solomon [31] peripherally mentions caregivers’ research responsibilities associated with the initial inquiry as well as the fact that they have in-facility information seeking interactions with IPCs. These findings from Solomon’s ethnographic study, though not a central part of his analysis, parallel some of our own research findings. Kangas’ [32] ethnographic research into the travel of Yemeni patients to other countries for private medical care confirms the centrality of family members in making the initial decision to access care abroad and their knowledge brokering roles. Kingsbury et al. [33] state that caregivers sometimes need to assume essential decision-making responsibilities and that unanticipated changes in medical tourists’ health status can force them to “navigate shifting boundaries of…[their] roles.” Two autobiographical narratives written by caregivers about their journeys abroad with medical tourists offer the strongest source of confirmation of the findings reported herein [27,28]. The authors of both narratives disclosed their active participation in all three of the roles identified here. Indeed, these former informal caregivers engaged in most of the responsibilities attributed to each of the knowledge broker, companion, and navigator roles (see [33] for greater analytic discussion of these narratives). With the findings of this article, we assist with putting discussion of caregiver activities and responsibilities from these other sources into context by explicitly considering the roles to which they are contributing.

The existing informal caregiving literature demonstrates that there are indeed commonalities between the roles filled by medical tourists’ caregivers and those adopted by other types of informal caregivers. Much of this literature is focused on the practice of informal caregiving in the home (e.g., [6,8,10,25,34]). One difference between these caregivers and those discussed in this article is the person who serves as a first point of contact and ongoing informational resource. It is the IPC for medical tourists’ caregivers, whereas homecare nurses often serve in this capacity for ‘conventional’ (i.e., local, non-transnational, long-term) informal caregivers in the home [35,36]. Furthermore, our findings reveal that medical tourists’ caregivers sometimes arrange lodging and tours and transfer documents to and from the destination country, whereas these are not common activities for non-transnational caregivers [8,34,37]. Differences such as these can be attributed to the specific context of care (i.e., local vs. transnational care and familiar vs. unfamiliar environment). The greatest overlaps between roles undertaken by medical tourists’ caregivers and other types of caregivers come in relation to non-context-dependent functions, such as facilitating information transfer and engaging in symptom monitoring. Many studies of informal caregiving in the home have found that informal caregivers in this setting play a pivotal role in transmitting details on changes in patients’ health status to formal health care providers, keeping logs of symptom changes, and other similar activities [11,37-39]. There are also cases in which the specific activities undertaken by medical tourists’ caregivers are different from those of conventional informal caregivers but the overall roles being enacted are identical. For example, finding a wheelchair to aid in a medical tourist’s mobility in an airport is likely done with the same intent as arranging for a patient’s accessible transit pick-up at home, a common activity undertaken by caregivers in other contexts [34,37]; in both cases the caregiver functions as a navigator.

### Limitations

This analysis has some limitations. First, using semi-structured phone interviews to obtain data has some intrinsic limitations. The linguistic diversity of participants, for example, was limited. The primary interviewer’s ability to speak only two languages with fluency, French and English, disqualified potential participants who were not fluent in either, although one Spanish-language interview was accommodated. Furthermore, unspoken insights that may have been gleaned through the interviews were missed because they were not conducted face-to-face. The reliability and affordability of phone and Skype interviews (see [40]) outweighed these concerns as conducting interviews using this medium is what enabled us to recruit such an international sample of participants.

Second, although interviewing IPCs is useful, it results in a second-hand perspective on medical tourists’ informal caregivers. We believe that the strengths of interviewing this group in the context of this study, particularly that they were able to comment on trends across many caregivers, outweighed this limitation. Every interview we conducted captured experiences with hundreds of caregivers, which was necessary to create the broad understanding that we sought to provide with this analysis. However, our analysis needs to be complemented with first-hand accounts from caregivers, and we intend on pursuing this in the next phases of our research. We believe that the limitations we have identified here are acceptable and do not significantly compromise the rigor of the study or analysis.

### Future research directions

Although we provide the first dedicated investigation of the roles of informal caregivers in medical tourism, this article contributes only a small part to our understanding of them. We have not measured the frequency with which each role is adopted, we have not assessed the spatiality or temporality of these roles, nor have we considered the effectiveness of these roles in ameliorating or maintaining patient health or wellness or even offsetting what is known as ‘caregiver burden’. These all serve as important directions for future research. For example, the collective impact of the stressors encountered by informal caregivers in the practice of care is referred to as caregiver burden [41-43]. Significant burden might lead to ‘caregiver burnout’ [41,44-46]. In this article, we show that the friends and family members who accompany medical tourists abroad are indeed filling informal caregiving roles and it would be valuable to determine if and how the activities they undertake in their capacities as knowledge brokers, companions, and navigators result in exposure to burden and ultimately burnout. This knowledge could then be used to assist in identifying interventions to offset that burden if it is found to exist. The caregiver group could benefit from, for example, the development of informational tools that anticipate and address the possibility of caregiver burnout. Although there has been some discussion of the need to create reliable, evidence-informed informational tools about medical tourism and enhance patients’ access to such sources [47,48], there has been no consideration of whether or not medical tourists’ informal caregivers could similarly benefit from enhanced access to credible information. Second, any demonstration of burden or burnout among this group would contribute new evidence to the health equity debates that exist around the practice of medical tourism that focus on discerning who ‘benefits’ and who ‘loses’ from the existence of this health services trade (see, for example, [21,49]). Although informal caregivers are not addressed in contemporary analyses of the health equity effects of medical tourism, they need to be incorporated into examinations of this subject.

An important area for future research pertains to our participant group: IPCs. An enhanced understanding of the scope and scale of IPCs’ roles and responsibilities as a whole is very much needed. Such knowledge is critical given that many aspects of informal caregivers’ roles hinge on the time, energy, and attentiveness of IPCs. Yet, there is not an adequate understanding of IPC training regarding interactions with caregivers, or the proportion of their time allocations or work tasks assigned to dealing with such interactions. Furthermore, we have shown that IPC and informal caregiver roles intersect at many points in a medical tourist’s journey. The implications of these intersections for the roles assumed by each group are unknown, such as the benefits and drawbacks for the health and wellbeing of the medical tourist. Such research would not only be important in increasing the knowledge of caregiver roles and responsibilities, it would also expand the existing knowledge of relationships between other medical tourism stakeholder groups. Dedicated attention to IPC roles and responsibilities could, for example, be used to create a schematic that demonstrates the intersection of the roles of IPCs, formal health care providers, other staff from the medical facility and from hospitality services such as hotel concierges and airline representatives, caregivers, and medical tourists.

## Conclusions

We found that the friends and family members who accompany medical tourists abroad engage in caregiving roles almost continuously while abroad. They facilitate and supplement the efforts of formal health care providers at medical tourism facilities to ensure the patient’s health and wellbeing and shoulder some of the responsibilities that might otherwise be assigned to patients. In this way, caregivers can act as both amplifiers and buffers: they amplify the efforts of the facility staff through facilitation and supplementation while buffering medical tourists from stresses stemming from responsibilities, anxieties, and discomforts. Although there are parallels between some of our findings about caregiver roles and those shared in the existing caregiving literature, the unique transnational care context of informal caregiving in medical tourism reveals activities and responsibilities assigned to particular roles that are specific to this particular care practice.

We believe that the knowledge that has been gleaned about a heretofore neglected medical tourism stakeholder group, namely patients’ informal caregivers, and the roles filled by members of this group provides valuable insight into how the medical tourism industry operates. Given the integral roles that friends and family members play (and particularly while they are abroad), the practice of medical tourism and thus the industry that supports it seems highly dependent on their unpaid care work. As with other informal caregivers, these individuals are effectively overlooked “shadow workers” [50] – unpaid, untrained, and largely unrecognized care providers - in what is often reported as a highly lucrative industry. We believe that adopting this critical perspective in light of the findings is essential to effectively address the question ‘who ultimately benefits from medical tourism?’ that is central to the health equity debates that surround the medical tourism industry. Although this question is far beyond the scope of this article, we do demonstrate the value of considering the roles that *every* stakeholder group plays in enabling this global health services practice. Without having such knowledge it becomes impossible to effectively determine the full scope of the health equity impacts of medical tourism, mitigate the negative ones, and enhance the positive ones, which is a key issue currently being discussed in research and policy circles [6,14,21,51,52].

## Endnote

^a^Informal caregivers to patients recovering from cosmetic or bariatric surgeries were sometimes noted to be problematic to patients’ wellbeing because they can become visibly distressed by a patient’s appearance. Therefore, some IPCs who worked at facilities specializing in these procedures advised medical tourists to travel unaccompanied. See [53] for further examination.

## Competing interests

The authors declared no potential conflicts of interest with respect to the research, authorship, and/or publication of this article.

## Authors’ contributions

VC conducted the interviews, led the coding, and played a leadership role in writing this article. VAC is lead investigator on the study that funded this research and developed the protocol. She contributed to all aspects of the study and was heavily involved in preparing this manuscript. JS and LT contributed input to the data collection instrument, participated actively in the face-to-face analysis meeting, and provided feedback on drafts of this paper. JS also aided in recruitment. All authors reviewed and approved the submitted manuscript.
